# Development of Self-Active Aging Index (S-AAI) among rural elderly in lower northern Thailand classified by age and gender

**DOI:** 10.1038/s41598-023-29788-2

**Published:** 2023-02-15

**Authors:** Orawan Keeratisiroj, Nithra Kitreerawutiwong, Sunsanee Mekrungrongwong

**Affiliations:** Faculty of Public Health, Naresuan University, Phitsanulok, 65000 Thailand

**Keywords:** Health care, Medical research

## Abstract

This study aimed to develop a Self-Active Aging Index (S-AAI) for the rural community of Thailand using the World Health Organization (WHO) framework, and score it according to age and gender. Overall, 1,098 elderly people were randomly selected. The self-reported questionnaires were categorized into three segments: health, participation, and security according to the WHO framework. An exploratory factor analysis was used to determine appropriate components. The S-AAI comprised 28 indicators and 9 factors: (1) mental/subjective health; (2) physical health; (3) health behavior and chronic disease; (4) vision and hearing; (5) oral health; (6) social participation; (7) stability in life; (8) financial stability; and (9) secure living. The overall S-AAI for all components was 0.65, with the index inversely proportional to age, but with no gender differences. The S-AAI is potentially Thailand's first multi-dimensional interactive aging assessment tool with a unique cultural context for rural areas. Although this tool is valid, it requires reliability testing.

## Introduction

Human life expectancy has been increasing across the globe, and it is estimated that between 2015 and 2050, the proportion of the world's population aged 60 and above will nearly double, from 12 to 22%. This was initially evident in high-income countries (such as Japan), but by 2050, two-thirds of the world's population over 60 is expected to live in low- and middle-income countries, with Thailand among the middle-income countries^1^. Thailand has been witnessing an aging society since 2005. The proportion of elderly people accounts for 10.3% of the country's population, and it is expected that the proportion of the elderly population will continue to increase. Population projections from Thailand show that by 2022, Thailand will become a fully aged society and by 2032, it will enter a super-aged society^2^.

In 2002, the World Health Organization (WHO) had proposed the concept of active aging, which focuses on promoting the quality of life of the elderly in terms of abilities, values, benefits, and potential. By definition, active aging is an appropriate process that leads to good health, social participation, and security to enhance the quality of life of the elderly^3^. In this study, the researchers used the term “active aging” in the context of the WHO^4^, which has broadened the perspective on health. It is of the view that health is everyone’s responsibility, not just healthcare workers given that everyone has the potential to take care of individual health. This concept supports the process of stepping into an aging society in every country.

Several countries, including Thailand, have developed tools to assess the active aging of older adults in the past decade^2,5–19^. The development of a tool based on the Active Aging Index in accordance with the framework of the WHO focuses on three areas: health, participation, and security, with an increase in indicators according to the context of the area.

Previous studies from abroad have mostly presented national benchmarking indices; for example, the European Center Vienna developed an “Active Aging Index” to assess the active aging of older adults among the 28 European countries^20^. Thailand developed an active aging index using big data collected from the 2011 National Statistical Office survey, so there are limitations in some indicators that may not be covered, such as oral health and nutritional status.

In addition, past studies have found that the potential aging index varies by context, region, and gender^6^. Therefore, this study aimed to develop a new index of active aging that is specific to rural areas where older adults can assess themselves. Additionally, this tool was used to assess the active aging of the elderly according to gender and age group.

## Methods

### Data source and sample

This study used data from our previous survey of 1,098 elderly people aged 60 and above from lower northern Thailand^21^. The sample size was sufficient for the factor analysis, according to general guidelines, under which a sample of 50 is considered as very poor, 100 as poor, 200 as fair, 300 as good, 500 as very good, and 1,000 as excellent^22^.

The participants were selected using multistage random sampling. The first step was clustered in nine provinces of lower northern Thailand by lottery covering five provinces: Phitsanulok, Uttaradit, Sukhothai, Kamphaeng Phet, and Tak. Second, random samples were collected to select the districts in each province. Finally, to obtain the proportion of the elderly cluster sampling in each district, we followed simple random sampling to determine the number of health-promoting hospitals (SDHPH) in each district. SDHPH electives from the family file system was used to reduce selection bias. The selection criteria were as follows: (1) being at least 60 years old, (2) living in the area for more than six months, (3) able to communicate, and (4) willingness to participate. The exclusion criterion was individuals with a history of mental disorders because they often had an increased level of perception of threat. The Institutional Review Board of Naresuan University approved this study ethically (COA No. 003/2018; IRB No. 1102/60). The study procedures were carried out in accordance with the Declaration of Helsinki. All participants were informed about the study, and they signed the consent form prior to participation. Data were collected using a cross-sectional design from February to June 2019, with five public health professionals trained as research assistants, to reduce collection bias. All data generated or analysed during this study are included in this published article [and its supplementary information files].

### Measures

The research tool for data collection was a structured interview questionnaire developed by the researchers based on the framework for the development of WHO's Active Aging Index^3^ and a previous study in Thailand^2,6,23,24^ by adjusting the indicators in all 3 components and 32 indicators, namely health, participation, and security, to match the context of the Thai rural area in the lower northern region. These indicators will be used to extract factors that include components in various aspects, as shown in Table 1. In addition, general information of the samples was collected to describe characteristics such as age, gender, religion, occupation, literacy, and number of family members, living style, and length of stay in the community. These tools were validated by three experts for content validity with Indexes of Item-Objective Congruence greater than 0.5 prior to data collection.

**Table 1:** Definition of indicators.

Indicators	Coding
Health	Health1–Health17
Health1: Subjective physical health (1 month ago)	0 = Very poor; 1 = Poor; 2 = Reasonable; 3 = Good; 4 = Very good
Health2: Visual ability	0 = Invisible (blind); 1 = Can't see clearly; 2 = See clearly with glasses; 3 = See clearly without glasses
Health3: Hearing ability	0 = Can't hear (deaf); 1 = Can't hear clearly; 2 = Hearing clearly with hearing aids; 3 = Hearing clearly without hearing aids
Health4: Barthel ADL index groups	0 = Bedside (0–4); 1 = Close to home (5–11); 2 = Social Addiction (12–20)
Health5: Functional ability groups	0 = Low (5–8); 1 = Medium (9–12); 2 = High (13–15)
Health6: Number of Chronic diseases	0 = 2 or more diseases; 1 = 1 disease; 2 = none
Health7: Psychological distress	0 = Regularly; 1 = Sometimes; 2 = Never
Health8: No Happiness	0 = Regularly; 1 = Sometimes; 2 = Never
Health9: Sleep problem	0 = Regularly; 1 = Sometimes; 2 = Never
Health10: Forgetfulness problem	0 = Regularly; 1 = Sometimes; 2 = Never
Health11: Number of teeth at least 20	0 = Less than 20 teeth; 1 = 20 teeth or more
Health12: Chewing or swallowing food problems	0 = Regularly; 1 = Sometimes; 2 = Never
Health13: Body mass index level	0 = Obesity level 3; 1 = Obesity level 2; 2 = Obesity level 1; 3 = Normal; 4 = Thin level 1; 5 = Thin level 2; 6 = Thin level 3
Health14: Exercise/physical activity (1 year ago)	0 = Never; 1 = Rarely (2–3 times/year); 2 = Sometimes (2–3 times/month); 3 = Often (2–3 times/week); 4 = Regularly (every day)
Health15: Smoking (1 year ago)	0 = Regularly (every day); 1 = Often (2–3 times/week); 2 = Sometimes (2–3 times/month); 3 = Rarely (2–3 times/year); 4 = Never
Health16: Alcohol drinking (1 year ago)	0 = Regularly (every day); 1 = Often (2–3 times/week); 2 = Sometimes (2–3 times/month); 3 = Rarely (2–3 times/year); 4 = Never
Health17: Annual Checkup	0 = No; 1 = Yes
Participation	Par1 – Par6
Par1: Working	0 = No; 1 = Yes
Par2: Marital status	0 = Single; 1 = Widow/Divorced/Separated; 2 = Married
Par3: Providing financial support to families	0 = No; 1 = Sometimes; 2 = Always
Par4: Being a group member or club	0 = No; 1 = Yes
Par5: Participation in the activities of the elderly club	0 = No; 1 = Sometimes; 2 = Always
Par6: Meeting neighbors or relatives	0 = Never; 1 = Rarely (2–3 times/year); 2 = Sometimes (2–3 times/month); 3 = Often (2–3 times/week); 4 = Regularly (every day)
Security	Sec1 – Sec9
Sec1: Housing ownership	0 = No; 1 = Yes
Sec2: Living status	0 = Relative/resident; 1 = Father or mother of the head of the household; 2 = Head of household or spouse of head of household
Sec3: Residential safety	0 = High risk; 1 = Low risk; 2 = Safety
Sec4: Education level	0 = Not studying; 1 = Elementary school (Grade 1–3); 2 = Elementary School (Grad 4 – 6); 3 = Middle School (Years 1- 3); 4 = High School (Year 4–6); 5 = Diploma/equivalent; 6 = Bachelor's degree or above
Sec5: Income level*	0 = No income; 1 = Less than 50,000 baht/year; 2 = 50,000–99,999 baht/year; 3 = 100,000 baht/year or more
Sec6: Main source of income	0 = Working; 1 = Pension; 2 = Pension for the elderly/disabled; 3 = Family subsidy; 4 = Savings and Investments
Sec7: Sufficiency of income	0 = No; 1 = Sometimes; 2 = Always
Sec8: Saving	0 = No; 1 = Yes
Sec9: Debt	0 = Yes 1 = No

*1 USD = 32.94 Thai Baht on January 31, 2023.

### Statistical analyses

Descriptive statistics, including frequency and percentage, were used to describe categorical data, whereas mean and standard deviation was used for continuous data of demographic characteristics. Exploratory factor analysis (EFA) was used to explore and group highly correlated items together to establish indicators that are key components of the policy framework for active aging by the WHO^3^. The EFA was run using SPSS version 17. Before conducting EFA, (1) z-scores for all items were generated because of variation between items, and (2) the Kaiser–Meyer–Olkin value was obtained to determine whether the sample was adequate; we also carried out Bartlett’s test of sphericity, which tests whether there is sufficient correlation between the variables and hence justifies the factor analysis. We then performed an EFA using (1) factor extraction using principal component analysis and (2) factor rotation using the Promax method. An eigenvalue greater than 1.0 is considered as a criterion to determine the number of components to be retained. S-AAI scores ranging from 0 to 1 were calculated for all participants by following the weight score for each dimension using raw scores^2,8,10,24^ (Supplementary Material). Descriptive statistics were calculated to obtain S-AAI scores by age and gender and the difference was tested by one-way analysis of variance and t-test for independence at the statistical level of 0.05.

### Informed consent

All participants were informed about the study, and they signed the informed consent form prior to participation.

## Result

### Sample characteristics

The sample consisted of 1,098 individuals with an average age of 71.01 (SD 7.55 years; age range 60–95). Most participants were women (61.4%) and Buddhists (98.5%). Before the age of 60, a majority of the participants had been farmers (65.4%), but after retirement, most were not engaged (50.5%), followed by farmers (35.6%). About half of the elderly participants could read (52.4%) and write (48.3%). Housing aspect: There were between 1–13 family members (Mean = 3.54, SD = 1.76). Nearly one in ten accounted for the elderly living alone (9.2%), with the elderly living with a husband/wife and their relatives (31.0%), children (35.2%), and spouses (24.5%) in comparable proportions. Most of them were residents of the community since birth (Mean 52.27 years, SD 18.53 years).

### Exploratory factor analysis (EFA) results

The indicators of the 32 variables in Table 1 were analyzed to extract factors according to the WHO concept in three components (health, participation, and security). The factor structure was examined using principal component extraction with Varimax rotation (n = 1,098). The Bartlett Sphericity test (p < 0.001) and Kaiser–Meyer–Olkin (KMO = 0.729) test indicated that factor analysis seemed to be highly adjusted for this analysis. Nine factors (Table 2) explained 55.373% of the total variance. A total of 28 retained items had factor loading values greater than 0.3 (four items with factor loadings lower than 0.3 were omitted), and only one of the nine factors could be described meaningfully in terms of respective components. The nine new factors of active aging were theoretically organized and presented in a simple structure. These factors were named after the WHO framework as follows: (1) mental/subjective health, (2) physical health, (3) health behavior and chronic disease, (4) vision and hearing, (5) oral health, (6) social participation, (7) stability in life, (8) financial stability, and (9) secure living.

**Table 2:** Structural and factor loading of active aging items among rural elderly (n = 1,098).

WHO framework	Factor	Factor loading	% of variance
Health	1. Mental/Subjective health		13.033
	No happiness (Health8)	0.866	
	Psychological distress (Health7)	0.846	
	Sleep problem (Health9)	0.598	
	Forgetfulness problem (Health10)	0.585	
	Subjective physical health (Health1)	0.434	
	2. Physical health		6.075
	Barthel ADL index groups (Health4)	0.736	
	Functional ability groups (Health5)	0.599	
	Exercise/physical activity (Health14)	0.576	
	3. Health behavior and chronic disease		5.878
	Smoking (Health15)	0.727	
	Alcohol drinking (Health16)	0.642	
	BMI level (Health13)	− 0.595	
	Number of Chronic disease (Health6)	0.531	
	4. Vision and hearing		3.962
	Hearing ability (Health3)	0.806	
	Visual ability (Health2)	0.778	
	5. Oral health		3.849
	Number of teeth at least 20 (Health11)	0.841	
	Chewing or swallowing food problems (Health12)	0.724	
Participation	6. Social participation		4.502
	Being a group member or club (Par4)	0.837	
	Participation in the activities of the elderly club (Par5)	0.682	
Security	7. Stability in life		7.814
	Working (Par1)	0.676	
	Main source of income (Sec6)	0.648	
	Debt (Sec9)	0.613	
	Income level (Sec5)	− 0.536	
	Education level (Sec4)	− 0.481	
	8. Financial stability		5.312
	Sufficiency of income (Sec7)	0.730	
	Saving (Sec8)	0.643	
	Providing financial support to families (Par3)	0.533	
	9. Secure living		4.950
	Living status (Sec2)	0.868	
	Housing ownership (Sec1)	0.839	
Total			55.373

Factor loadings lower than 0.3 were omitted (Health17, Par2, Par6, Sec3).

### Self-Active Aging Index (S-AAI) results

**Figure 1:**
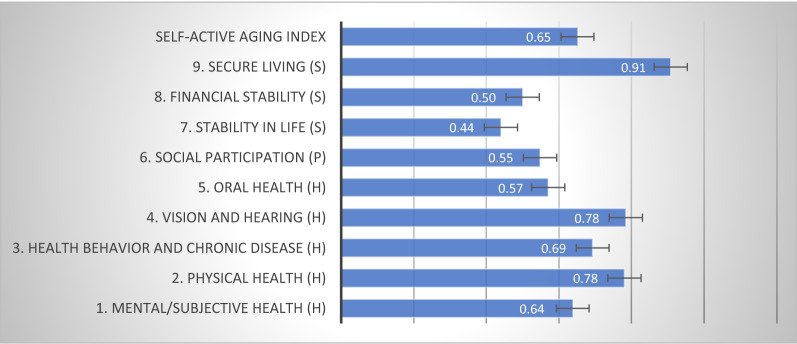
S-AAI scores.

The S-AAI scores for elderly individuals in rural Thailand for each factor is shown in Fig. 1. The mean overall S-AAI score was 0.65. Secure living (security domain) showed the highest score (0.91), followed by health: vision and hearing (0.78) and physical health (0.78). For other domains, the score was less than 0.70 (Fig. 1).

**Figure 2:**
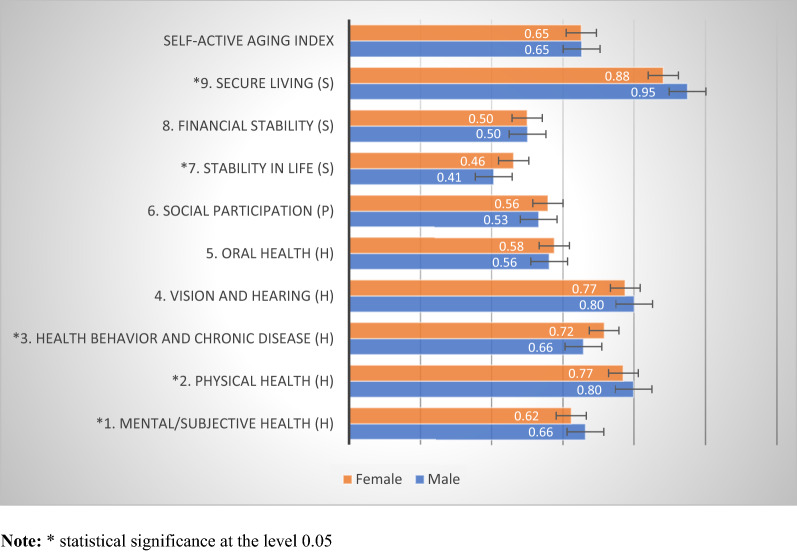
S-AAI scores classified by gender.

**Figure 3:**
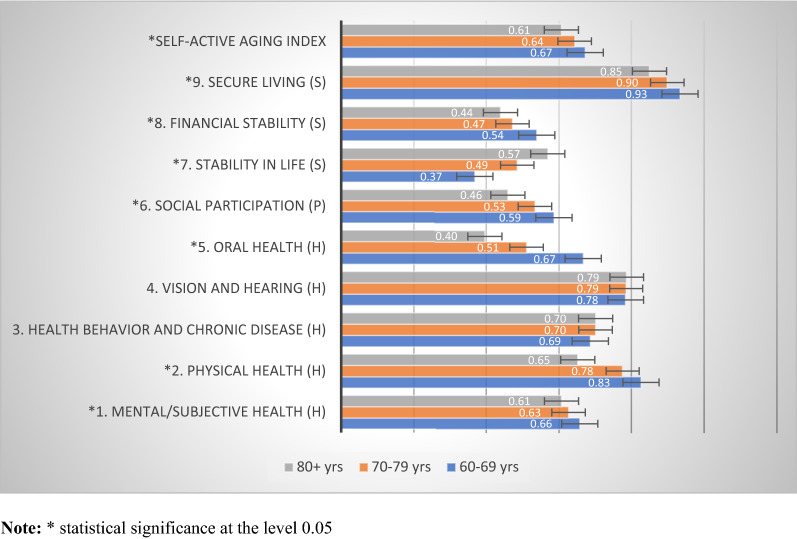
S-AAI scores classified by age groups.

Figure 2 shows the distribution of overall S-AAI and domain scores stratified by gender. The S-AAI was 0.65 for both male and female elderly individuals. When considering each domain, it was found that elderly males scored significantly higher than females in terms of mental/subjective health, physical health, and secure living. In contrast, older women scored higher than men for health behavior, chronic disease, and stability in life. In addition, Fig. 3 shows the classification of S-AAI scores by age group, which found that the scores were statistically significantly inversely related to age, with the age group 60–69 having the highest score (0.67), followed by those aged 70–79 (0.64), and 80 and over (0.61). When considering each domain, it was found that the S-AAI scores differed statistically, with the scores decreasing with increasing age for mental/subjective health, physical health, oral health, social participation, financial stability, and secure living, while it increased with age for stability in life.

## Discussion

This study aimed to develop an S-AAI for the rural elderly population in Thailand. This tool was developed based on the WHO conceptual framework (health, participation, and security) and a review of 32 indicators of active aging in Thailand. After exploratory factor analysis of the remaining 28 indicators, they were categorized into nine determinant factors that were similar to the previous study^18,19^ and a systematic review of the diverse and varied facets of active aging comprises 15 topics^25^. However, the employment component that is not included in the S-AAI differs from the international index development^15^. Considering the working context of Thai elderly in rural areas, most of them are farmers or have families who take care of them. This index mostly focuses on health components, comprising five factors and sixteen indicators. Personal health is an essential condition of independent living in relation to the individual, family, and social environment^3^.

The first major determinant of active aging is mental/subjective health, which explains the greatest variability in S-AAI. This factor includes five indicators: no happiness, psychological distress, sleep problems, forgetfulness, and subjective physical health. Physical health (Factor 2) incorporated three items: the Barthel ADL Index, functional ability, and exercise/physical activity. Two of these health factors were of prime importance, indicating that older adults living a healthy physical and mental life with a perception of their own wellness experienced more active aging. The findings are consistent with numerous studies suggesting that a healthy lifestyle is closely related to healthy aging^23,26^ and successful aging^9^. This was previously confirmed by research that psychology has no strong relevance to aging^12,27^. In addition, a study in Brazil indicated that self-assessed health is an important health measure^28^. Factor 3 (health behavior and chronic disease) is related to health behavior and consists of four indicators: smoking, alcohol drinking, BMI level, and the number of chronic diseases. This is supported by several studies that have focused on health behaviors^6,18^. Vision and hearing (Factor 4) consisted of two indicators: hearing and visual abilities. Both indicators are determinants of the apparent health of the elderly owing to age-related deterioration. There is also a strong correlation between hearing impairment and loneliness in older adults^29^. Two indicators of oral health (Factor 5) were included: having at least 20 teeth and facing problems chewing or swallowing food, which were included based on recommendations from previous studies^18^.

The second component is participation, which consists of one factor and two indicators. The WHO defines participation as engagement in family, community, and social activities. These activities help create a sense of belonging, dignity, and emotional attachment to the family, which improves the mental and physical health of the elderly^3^. Our tool focusing on social participation (Factor 6) consisted of two items: being a group member or club and participation in activities of the elderly club. Some of the indicators based on the initial conceptual framework such as marital status and meeting neighbors or relatives were excluded. Additionally, some indicators, including working and providing financial support to families, were included in the security component.

The last component is security, which consists of 3 factors and 10 indicators. In this situation, having security guarantees means physical security and economic stability, giving the elderly a good quality of life^3^. Stability in life (Factor7) has also been identified as an important aspect of active aging. This factor involves work, the main source of income, debt, income level, and education level. According to the initial conceptual framework, the work was organized into a component related to participation. It also represents income that leads to stability in life. The three items loaded on Factor 8 (financial stability) consisted of sufficiency of income, savings, and providing financial support to families. Several studies have indicated that financial stability is important^5,6,12,18^. Financial security maximizes a sense of security and independence, especially among older adults, who are mostly unemployed. The last factor in active aging, which we call “secure living” (Factor 9), consists of two items: living status and housing ownership. The ingrained socio-cultural nature of rural Thailand promotes values ​​such as caring for and respecting the elderly^30^. The study found that more than 90 percent of the elderly had their own homes and did not live alone. Therefore, these two indicators are important for the identification of safety of the elderly in rural Thailand.

The nine factors developed the S-AAI score of the elderly in rural lower northern Thailand, indicating that their scores were moderate (0.65), similar to other studies that use large data at the national level in Thailand^2,18,24^ and other local studies^8,10^. There was no difference between genders in our study, which was inconsistent with other studies in Thailand^2,18,24^ and in several European countries^31^ that found that male elderly individuals had a higher index than their female counterparts. However, we found gender differences in some factors of elderly males had better active aging scores than females, including mental/subjective health, physical health, and secure living. In contrast, older women scored higher than men in terms of health behaviors, chronic diseases, and stability in life. Our data highlight the extreme differences between factors in the security component, with older adults in rural Thailand having the most secure lives (0.91), whereas stability in life (0.44) and financial stability (0.50) scored the lowest. The classification of scores by age group showed that the elderly tended to decline in scores, which was particularly evident in health conditions, except for the stability of life of the elderly in rural Thailand, which increased with age. These findings are unique to rural communities in Thailand. There is evidence to support differences between urban and rural communities in other countries^32^. This highlights the need to consider the variations in active aging in different regions of Thailand, as each area has different cultural characteristics^6^.

## Conclusion

In summary, health potential is the first condition for independent and self-reliant living, which is a key characteristic of older adults. As for the elderly's continued participation in activities, it not only gives them a sense of self-esteem but also a perception of dignity and psychological dependency of their family. These factors help to improve the mental health of the elderly and affect their physical health. In addition, various factors of security in life also helps the elderly experience physical security from safe living and economic security that gives them confidence to lead a quality life. The results of this study indicate that elderly people in rural areas in the lower northern Thailand have a moderate active aging index. Although there are some problems with financial stability, there is government support in terms of both pension and health insurance.

### Implications

The S-AAI tool can be used as a quantitative measure for older adults in rural communities to self-assess active aging, and potentially used in questionnaires or interviews in research and practice. Local administrators and related officials can apply the indicators that are the main components of the S-AAI for the rural elderly as a guideline for planning to improve the quality of life of the elderly. Finally, this tool can be developed further to make it more widely available. The new tool can be used to monitor active ageing outcomes at the country level and to investigate the potential of older people regarding actively participate in health and social activity to promote an active role for the older people.

### Strengths and limitations

The present study has some limitations. First, although the sample was large, it was not nationally representative, and other areas of Thailand may have different indicators of active aging. Therefore, further studies are needed to externally validate assessment tools developed in various cultural contexts. Second, as this study focuses on rural Thai culture, it cannot be generalized in other countries that may have different cultural contexts regarding certain S-AAI indicators such as housing and social activities, health services, etc. Third, due to the different environments in urban and rural areas, there is diversity in their economic structure, social participation, and demographics in the local community. Accordingly, the developed measuring tool may not be the same for older people in urban areas. Therefore, the study was limited to the development of measurement instruments for rural communities. Fourth, there are 5 research assistants to collect data, which may affect the data, especially the interviews with the older person who cannot read and write. However, the researcher has already controlled the quality of the data by training the research assistants prior to the fieldwork of data collection regarding the overview and objective of the research and the research techniques until they achieved research skills in data collection. Lastly, the study has not examined the psychometric properties of the S-AAI used for reliability, which is our next study, including confirmation factor analysis.

The strength of this study is that the indicators of active aging at the individual level were used comprehensively in many dimensions as the foundation for further development. The novelty of some indicators that are important in the elderly but have not yet been compiled in Thailand, namely oral health and nutritional status, were included in this study. Finally, the development of a scale for active aging assessment is a quantitative structure that allows participants to self-assess.

## Supplementary Information

Supplementary Information 1.

Supplementary Information 2.

Supplementary Information 3.

Supplementary Information 4.

## Data Availability

All data generated or analysed during this study are included in this published article [and its supplementary information files].
