# Supplementary material for: Development of Self-Active Aging Index (S-AAI) among rural elderly in lower northern Thailand classified by age and gender
Source: Sci Rep. 2023 Feb 15;13:2676. doi: 10.1038/s41598-023-29788-2 (PMC9932059; doi:10.1038/s41598-023-29788-2)
Supplement: Supplementary file 1 — Supplementary Information 1. [file 41598_2023_29788_MOESM1_ESM.pdf]

Supplementary material-S1

The calculation for Self-Active Aging Index (S-AAI) followed by formula:

Index = $\sum_{i=1}^{n}\left(\frac{\bar{x}_i}{M_i x n}\right)$, by

$\bar{x}_i$ = Mean of indicator i

M_i = Maximum of the value of indicator i

n = Number of indicators for dimension

F1 - Mental/subjective health

$$\frac{\text{No happiness}}{2x5}+\frac{\text{Psychological distress}}{2x5}+\frac{\text{Subjective physical health}}{4x5}+\frac{\text{Sleep problem}}{2x5}+\frac{\text{Forgetfulness problem}}{2x5}+\frac{\text{Subjective physical health}}{4x5}$$

F2 - Physical health

$$\frac{\text{Barthel ADL index groups}}{2x3}+\frac{\text{Functional ability groups}}{2x3}+\frac{\text{Exercise or physical activity}}{4x3}$$

F3 -Health behavior and chronic disease

$$\frac{\text{Smoking}}{4x4}+\frac{\text{Alcohol drinking}}{4x4}+\frac{\text{BMI level}}{6x4}+\frac{\text{Number of Chronic disease}}{2x4}$$

F4 -Vision and hearing

$$\frac{\text{Hearing ability}}{3x2}+\frac{\text{Visual ability}}{3x2}$$

F5 -Oral health

$$\frac{\text{Number of teeth at least 20}}{1x2}+\frac{\text{Chewing or swallowing food problems}}{2x2}$$

F6 -Social participation

$$\frac{\text{Being a group member or club}}{\mathbf{1}\boldsymbol{x}\mathbf{2}} + \frac{\text{Participation in the activities of the elderly club}}{\mathbf{2}\boldsymbol{x}\mathbf{2}}$$

F7 -Stability in life

$$\frac{\text{Working}}{\mathbf{1}\boldsymbol{x}\mathbf{5}} + \frac{\text{Main source of income}}{\mathbf{4}\boldsymbol{x}\mathbf{5}} + \frac{\text{Debt}}{\mathbf{1}\boldsymbol{x}\mathbf{5}} + \frac{\text{Income level}}{\mathbf{3}\boldsymbol{x}\mathbf{5}} + \frac{\text{Education level}}{\mathbf{6}\boldsymbol{x}\mathbf{5}}$$

F8 -Financial stability

$$\frac{\text{Sufficiency of income}}{\mathbf{2}\boldsymbol{x}\mathbf{3}} + \frac{\text{Saving}}{\mathbf{1}\boldsymbol{x}\mathbf{3}} + \frac{\text{Providing financial support to families}}{\mathbf{2}\boldsymbol{x}\mathbf{3}}$$

F9 -Secure living

$$\frac{\text{Living status}}{\mathbf{2}\boldsymbol{x}\mathbf{2}} + \frac{\text{Housing ownership}}{\mathbf{1}\boldsymbol{x}\mathbf{2}}$$

Total S-AAI score

$$\boldsymbol{S - AAI} = \frac{\boldsymbol{F1 + F2 + F3 + F4 + F5 + F6 + F7 + F8 + F9}}{\mathbf{9}}$$
