# Supplementary material for: Development of Self-Active Aging Index (S-AAI) among rural elderly in lower northern Thailand classified by age and gender
Source: Sci Rep. 2023 Feb 15;13:2676. doi: 10.1038/s41598-023-29788-2 (PMC9932059; doi:10.1038/s41598-023-29788-2)
Supplement: Supplementary file 2 — Supplementary Information 2. [file 41598_2023_29788_MOESM2_ESM.pdf]

Supplementary material-S2

Table 1 Self-Active Aging Index (S-AAI) score and its 95% confidence interval.

**Factors**	**S-AAI score (n = 1,098)**	
	**Mean ± SD**	**95% CI**
1. Mental/Subjective health	0.64 ± 0.18	0.63 – 0.65
2. Physical health	0.78 ± 0.20	0.77 – 0.79
3. Health behavior and chronic disease	0.69 ± 0.14	0.68 – 0.70
4. Vision and hearing	0.78 ± 0.23	0.77 – 0.80
5. Oral health	0.57 ± 0.33	0.55 – 0.59
6. Social participation	0.55 ± 0.34	0.53 – 0.57
7. Stability in life	0.44 ± 0.17	0.43 – 0.45
8. Financial stability	0.50 ± 0.27	0.48 – 0.52
9. Secure living	0.91 ± 0.23	0.89 – 0.92
Total	0.65 ± 0.10	0.65 – 0.66

Table 2 Self-Active Aging Index (S-AAI) score and its 95% confidence interval classified by gender.

**Factors**	**S-AAI score for male (n = 424)**		**S-AAI score for female (n = 674)**	
	**Mean ± SD**	**95% CI**	**Mean ± SD**	**95% CI**
1. Mental/Subjective health	0.66 ± 0.18	0.65 – 0.68	0.62 ± 0.17	0.61 – 0.64
2. Physical health	0.80 ± 0.19	0.78 – 0.82	0.77 ± 0.20	0.75 – 0.78
3. Health behavior and chronic disease	0.66 ± 0.16	0.64 – 0.67	0.72 ± 0.12	0.71 – 0.72
4. Vision and hearing	0.80 ± 0.22	0.78 – 0.82	0.77 ± 0.23	0.76 – 0.79
5. Oral health	0.56 ± 0.33	0.53 – 0.59	0.58 ± 0.45	0.55 – 0.60
6. Social participation	0.53 ± 0.34	0.50 – 0.56	0.56 ± 0.34	0.53 – 0.58
7. Stability in life	0.41 ± 0.17	0.39 – 0.42	0.46 ± 0.17	0.45 – 0.47
8. Financial stability	0.50 ± 0.27	0.47 – 0.53	0.50 ± 0.27	0.48 – 0.52
9. Secure living	0.95 ± 0.17	0.93 – 0.97	0.88 ± 0.25	0.86 – 0.90
Total	0.65 ± 0.10	0.64 – 0.66	0.65 ± 0.11	0.65 – 0.66

Table 3 Self-Active Aging Index (S-AAI) score and its 95% confidence interval classified by age groups.

**Factors**	**S-AAI score for 60 – 69 yrs (n = 550)**		**S-AAI score for 70 – 79 yrs (n = 370)**		**S-AAI score for 80+ yrs (n = 178)**	
	**Mean ± SD**	**95% CI**	**Mean ± SD**	**95% CI**	**Mean ± SD**	**95% CI**
1. Mental/Subjective health	0.66 ± 0.18	0.64 – 0.67	0.63 ± 0.17	0.61 – 0.64	0.61 ± 0.18	0.58 – 0.63
2. Physical health	0.83 ± 0.16	0.81 – 0.84	0.78 ± 0.20	0.75 – 0.80	0.65 ± 0.24	0.61 – 0.69
3. Health behavior and chronic disease	0.69 ± 0.15	0.67 – 0.70	0.70 ± 0.13	0.69 – 0.71	0.70 ± 0.13	0.68 – 0.72
4. Vision and hearing	0.78 ± 0.24	0.76 – 0.80	0.79 ± 0.22	0.76 – 0.81	0.79 ± 0.22	0.75 – 0.82
5. Oral health	0.67 ± 0.32	0.64 – 0.69	0.51 ± 0.31	0.48 – 0.54	0.40 ± 0.29	0.35 – 0.44
6. Social participation	0.59 ± 0.33	0.56 – 0.61	0.53 ± 0.34	0.50 – 0.57	0.46 ± 0.36	0.41 – 0.51
7. Stability in life	0.37 ± 0.17	0.35 – 0.38	0.49 ± 0.16	0.47 – 0.50	0.57 ± 0.10	0.55 – 0.58
8. Financial stability	0.54 ± 0.27	0.52 – 0.56	0.47 ± 0.28	0.44 – 0.50	0.44 ± 0.23	0.40 – 0.47
9. Secure living	0.93 ± 0.20	0.92 – 0.95	0.90 ± 0.23	0.87 – 0.92	0.85 ± 0.28	0.81 – 0.92
Total	0.67 ± 0.09	0.66 – 0.68	0.64 ± 0.09	0.63 – 0.65	0.61 ± 0.09	0.59 – 0.62
