# Supplementary material for: Development of Self-Active Aging Index (S-AAI) among rural elderly in lower northern Thailand classified by age and gender
Source: Sci Rep. 2023 Feb 15;13:2676. doi: 10.1038/s41598-023-29788-2 (PMC9932059; doi:10.1038/s41598-023-29788-2)
Supplement: Supplementary file 3 — Supplementary Information 3. [file 41598_2023_29788_MOESM3_ESM.pdf]

Supplementary material-S3

Table 1 Descriptive statistics for health indicators (n = 1,098).

**Health Indicators (Health1 - Health17)**		**n (%)**
Health1	Subjective physical health (1 month ago)	
	Very poor	30 (2.7)
	Poor	410 (37.3)
	Reasonable	487 (44.4)
	Good	124 (11.3)
	Very good	47 (4.3)
Health2	Visual ability	
	Invisible (blind)	9 (0.8)
	Can't see clearly	220 (20.0)
	See clearly with glasses	368 (33.5)
	See clearly without glasses	501 (45.6)
Health3	Hearing ability	
	Can't hear (deaf)	13 (1.2)
	Can't hear clearly	232 (21.1)
	Hearing clearly with hearing aids	80 (7.3)
	Hearing clearly without hearing aids	773 (70.4)
Health4	Barthel ADL index groups	
	Bedside (0-4)	8 (0.7)
	Close to home (5-11)	20 (1.8)
	Social Addiction (12-20)	1,070 (97.4)
Health5	Functional ability groups	

Health Indicators (Health1 - Health17)		n (%)
	Low (5-8)	134 (12.2)
	Medium (9-12)	221 (21.1)
	High (13-15)	743 (67.7)
Health6	Number of Chronic diseases	
	2 or more diseases	328 (29.9)
	1 disease	411 (37.4)
	none	359 (32.7)
Health7	Psychological distress	
	Regularly	31 (2.8)
	Sometimes	417 (38.0)
	Never	650 (59.2)
Health8	No Happiness	
	Regularly	35 (3.2)
	Sometimes	502 (45.7)
	Never	561 (51.1)
Health9	Sleep problem	
	Regularly	45 (4.1)
	Sometimes	723 (65.8)
	Never	330 (30.1)
Health10	Forgetfulness problem	
	Regularly	55 (5.0)
	Sometimes	773 (70.4)
	Never	270 (24.6)

Health Indicators (Health1 - Health17)		n (%)
Health11	Number of teeth at least 20	
	Less than 20 teeth	565 (51.5)
	20 teeth or more	533 (48.5)
Health12	Chewing or swallowing food problems	
	Regularly	85 (7.7)
	Sometimes	588 (53.6)
	Never	425 (38.7)
Health13	Body mass index level	
	Obesity level 3	2 (0.2)
	Obesity level 2	46 (4.2)
	Obesity level 1	222 (20.2)
	Normal	678 (61.7)
	Thin level 1	79 (7.2)
	Thin level 2	29 (2.6)
	Thin level 3	42 (3.8)
	Mean ± SD	22.66 ± 4.04
	Median (Min-Max)	22.33 (12.49 –41.15)
Health14	Exercise/physical activity (1 year ago)	
	Never	140 (12.8)
	Rarely (2-3 times/year)	145 (13.2)
	Sometimes (2-3 times/month)	278 (25.3)
	Often (2-3 times/week)	286 (26.0)
	Regularly (every day)	249 (22.7)

Health Indicators (Health1 - Health17)		n (%)
Health15	Smoking (1 year ago)	
	Regularly (every day)	125 (11.4)
	Often (2-3 times/week)	39 (3.6)
	Sometimes (2-3 times/month)	10 (0.9)
	Rarely (2-3 times/year)	15 (1.4)
	Never	909 (82.8)
Health16	Alcohol drinking (1 year ago)	
	Regularly (every day)	30 (2.7)
	Often (2-3 times/week)	41 (3.7)
	Sometimes (2-3 times/month)	45 (4.1)
	Rarely (2-3 times/year)	38 (3.5)
	Never	944 (86.0)
Health17	Annual Checkup	
	No	102 (9.3)
	Yes	996 (90.7)

Table 2 Descriptive statistics for participation indicators (n = 1,098).

**Participation indicators (Par1 – Par6)**		**n (%)**
Par1	Working	
	No	555 (50.5)
	Yes	543 (49.5)
Par2	Marital status	
	Single	40 (3.6)
	Widow/Divorced/Separated	636 (57.9)
	Married	422 (38.4)
Par3	Providing financial support to families	
	No	140 (12.8)
	Sometimes	464 (42.3)
	Always	494 (45.0)
Par4	Being a group member or club	
	No	413 (37.6)
	Yes	685 (62.4)
Par5	Participation in the activities of the elderly club	
	No	247 (22.5)
	Sometimes	666 (60.7)
	Always	185 (16.8)
Par6	Meeting neighbors or relatives	
	Never	71 (6.5)
	Rarely (2-3 times/year)	165 (15.0)
	Sometimes (2-3 times/month)	243 (22.1)

**Participation indicators (Par1 – Par6)**		**n (%)**
	Often (2-3 times/week)	337 (30.7)
	Regularly (every day)	282 (25.7)

Table 3 Descriptive statistics for security indicators (n = 1,098).

**Security indicators (Sec1 – Sec9)**		**n (%)**
Sec1	Housing ownership	
	No	93 (8.5)
	Yes	1,005 (91.5)
Sec2	Living status	
	Relative/resident	51 (4.6)
	Father or mother of the head of the household	115 (10.5)
	Head of household or spouse of head of household	932 (84.9)
Sec3	Residential safety	
	High risk	36 (3.3)
	Low risk	85 (7.8)
	Safety	969 (88.9)
Sec4	Education level	
	Not studying	185 (16.8)
	Elementary school (Grade 1 - 3)	123 (11.2)
	Elementary School (Grad 4 – 6)	708 (64.5)
	Middle School (Years 1- 3)	24 (2.2)
	High School (Year 4 - 6)	35 (3.2)
	Diploma/equivalent	5 (0.5)
	Bachelor's degree or above	18 (1.6)
Sec5	Income level*	
	No income	90 (8.2)
	Less than 50,000 baht/year	855 (77.9)

Security indicators (Sec1 – Sec9)		n (%)
	50,000 – 99,999 baht/year	116 (10.6)
	100,000 baht/year or more	37 (3.4)
Sec6	Main source of income	
	Working	201 (18.3)
	Pension	41 (3.7)
	Pension for the elderly/disabled	740 (67.4)
	Family subsidy	92 (8.4)
	Savings and Investments	24 (2.2)
	Pension for the elderly/disabled (Bath/month)*	
	Mean ± SD	755.52 ± 266.99
	Median (Min – Max)	700 (600-1800)
Sec7	Sufficiency of income	
	No	329 (30.0)
	Sometimes	541 (49.3)
	Always	228 (20.8)
Sec8	Saving(Bath) *	
	No	675 (61.5)
	Yes	423 (38.5)
	Mean ± SD	29,319.9 ± 79,419.73
	Median (Min – Max)	10,000 (200-1,000,000)
Sec9	Debt (Bath) *	
	Yes	422 (38.4)
	No	676 (61.6)

Security indicators (Sec1 – Sec9)		n (%)
	Mean ± SD	144,871 ± 385,553
	Median (Min – Max)	50,000 (500-5,000,000)

Note * 1 USD = 32.94 Thai Baht on January 31, 2023
